# Electronic Excitation Dynamics in Liquid Water under Proton Irradiation

**DOI:** 10.1038/srep40379

**Published:** 2017-01-13

**Authors:** Kyle G. Reeves, Yosuke Kanai

**Affiliations:** 1 Department of Chemistry, University of North Carolina at Chapel Hill, Chapel Hill, North Carolina 27599, USA

## Abstract

Molecular behaviour of liquid water under proton irradiation is of great importance to a number of technological and medical applications. The highly energetic proton generates a time-varying field that is highly localized and heterogeneous at the molecular scale, and massive electronic excitations are produced as a result of the field-matter interaction. Using first-principles quantum dynamics simulations, we reveal details of how electrons are dynamically excited through non-equilibrium energy transfer from highly energetic protons in liquid water on the atto/femto-second time scale. Water molecules along the path of the energetic proton undergo ionization at individual molecular level, and the excitation primarily derives from lone pair electrons on the oxygen atom of water molecules. A reduced charge state on the energetic proton in the condensed phase of water results in the strongly suppressed electronic response when compared to water molecules in the gas phase. These molecular-level findings provide important insights into understanding the water radiolysis process under proton irradiation.

The use of ion beams for treating cancer was first conceptualized in 1946^1^. Today, the use of highly energetic protons for cancer therapy has become increasingly more popular, and more than a few dozen ion beam therapy facilities have been constructed worldwide^2^,^3^. The use of charged-particle radiation such as fast, energetic protons over more conventional radiation based on photons or electrons is often considered more attractive because of its distinct energy deposition profile^4^,^5^. This so-called Bragg curve characterizes the energy loss of ionizing radiation as it penetrates through matter. A distinct feature of using ion radiation for the treatment is that the characteristic Bragg curve exhibits a very sharp peak, called the Bragg peak. In the context of proton beam therapy, one would calibrate the initial kinetic energy of the protons such that this sharp Bragg peak would be located at a depth corresponding to the location of the tumour.

At a scientific level, the Bragg curve profile is determined by the electronic stopping power (also known as unrestricted linear energy transfer), which measures the rate of energy transfer from the charged particle to electrons in matter per unit distance of the energetic particle’s movement^6^,^7^,^8^,^9^,^10^. The stopping power is a continuous function of the particle velocity (or kinetic energy), and the velocities nearest to the maximum of the stopping power are responsible for the formation of the sharp Bragg peak. Because liquid water is the majority constituent in human cells, various models for the electronic stopping power in liquid water have been proposed over the years^11^. At the same time, such analytical models of the electronic stopping power provide minimal information in terms of what is actually happening at the molecular level. In electronic stopping, the electrons in the liquid water are excited by the fast ion through a non-equilibrium energy transfer process^12^. For molecular liquids such as water, molecular details of the excitation process are crucial for developing a better understanding of the radiation-induced cancer therapy that utilizes proton and other ion irradiations^13^. For example, work by Montenegro *et al*. on water fragmentation from the electronic stopping of carbon ions in liquid water shows that yields of fragmented radicals do not obey the stopping power curve (as a function of the ion kinetic energy)^14^. The development of a molecular-level understanding of the electronic stopping in the liquid water is fundamental to ultimately explain how ion radiation induces highly irreparable DNA double strand break (DSB) damage, a central event in cancer therapies^15^,^16^. In principle, ion radiation can induce DSB damage directly within DNA or indirectly via water radiolysis, which produces highly reactive radicals (Fig. 1) 17,18. Understanding how the proton irradiation produces the electronic excitation in liquid water at the molecular level is the first step towards understanding the extent to which the direct and indirect mechanisms are at play, especially in comparison to the more conventional photo-induced electronic excitation in liquid water^19^,^20^,^21^. Here, we present a molecular-level description of the electronic excitation dynamics in liquid water under proton irradiation via first-principles theory. Using non-equilibrium electron dynamics simulations based on our new large-scale real-time time-dependent density functional theory approach^22^,^23^, key features in the electronic excitation are revealed at both mesoscopic and molecular levels for deciphering the water radiolysis mechanism under proton irradiation.

## Results and Discussion

The dynamical response of electrons to the energetic proton is simulated using a real-time time-dependent density functional theory (RT-TDDFT) approach as discussed in the Methods section, and technical details of how this non-equilibrium quantum dynamics simulations can be used to study electronic stopping process is discussed in our recent work^24^. Briefly, the simulation contains 162 water molecules with periodic boundary conditions. The equilibrium structure of the liquid water molecules at room temperature was prepared by performing a separate first-principles molecular dynamics simulation. Since we are interested in the excitation dynamics near the Bragg peak, we first identify the maximum electronic response as a function of the proton kinetic energy for a specific proton path. The magnitude of the non-adiabatic force on the energetic proton and the single-particle excitation probability are shown in Fig. 2, and they were used to identify the specific proton kinetic energy of interest for detailed analysis. The work done by the non-adiabatic force on the energetic proton manifests as the electronic excitation^25^, and the non-adiabatic force is equivalent to instantaneous electronic stopping power^26^. Single-particle excitation probability (SEP) is defined here as time-dependent change of the total occupation in valence band electronic states of the extended system of liquid water at electronic equilibrium. While the non-adiabatic forces and SEP as a function of kinetic energy follow roughly the same trend (see Fig. 2), SEP is not directly related to the instantaneous electronic stopping power. For the specific proton path we investigate here, the proton kinetic energy of ~20 keV (velocity of ~0.9 a.u) yields the greatest electronic response. Although an experimental stopping power curve for liquid water does not exist near its maximum, various analytical models predict that the maximum energy dissipation occurs around 10 keV~100 keV^11^. Our simulations are also consistent with these predictions such that the ensemble averaged electronic stopping power curve shows a peak in this kinetic energy range as discussed in our recent work^24^. In the following, we will discuss the excitation dynamics in liquid water at this proton kinetic energy, which exhibits the maximum electronic response for the proton path.

In the time that it takes for the energetic proton to pass through the simulation cell in approximately one femtosecond (see Methods), the positions of the water molecules remain essentially unchanged. Thus, it is possible to analyse the time-evolving, non-equilibrium electron density in terms of single-particle transitions between occupied and unoccupied electronic states at equilibrium (i.e. t = 0 fs). For an extended system like liquid water, it is convenient to project the time-dependent wavefunctions onto the eigenstates of the equilibrium electron density so that the electronic excitation dynamics can be characterized in the framework of the single-particle excitations. We visualize the changes in electron density associated with the excitations by separating the contributions from originally occupied and unoccupied states at equilibrium (see Methods). Figure 3 shows the excitation behaviour at three different instants in time. The projection onto the occupied eigenstates show that electrons are excited away from the proton track (Fig. 3a–c) while the projection onto the unoccupied eigenstates show that the electrons are excited onto the entire liquid water (Fig. 3d–f). The water molecules along the proton path (within 2 Å) effectively undergo ionization at the individual molecular level. This excitation behaviour is distinctively different from what we previously observed for metals^22^,^25^ for which the electric field generated by the energetic proton is highly screened, and the electronic excitation was thus rather confined along the proton track.

**Figure 1:**
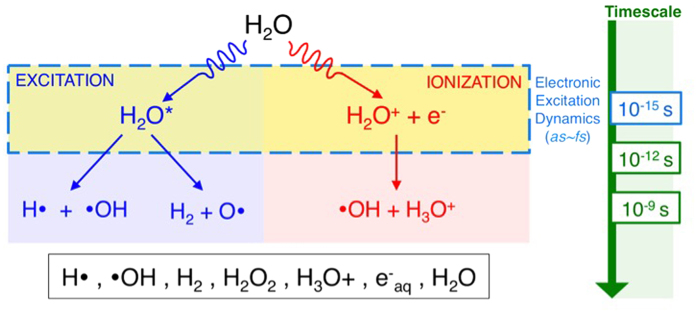
Schematic of mechanisms of radical generation in water radiolysis. Radical formation in water is generally believed to occur through two mechanisms: excitation or ionization at the individual molecular level.

**Figure 2:**
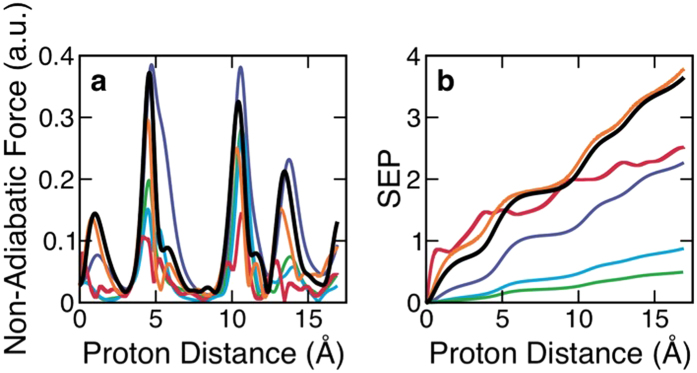
Velocity-dependent electronic response in liquid water to energetic proton. (**a**) Non-adiabatic force exerted on the energetic proton as it travels through liquid water. (**b**) Single-particle excitation probability (SEP) in response to the energetic proton’s movement in liquid water. Peaks in the non-adiabatic force curves correspond approximately to regions where the proton comes close to water molecules. In both plots, the equivalent proton kinetic energies are 5 keV (red), 10 keV (orange), 20 kev (black), 100 keV (purple), 500 keV (blue), and 1 MeV (green).

**Figure 3:**
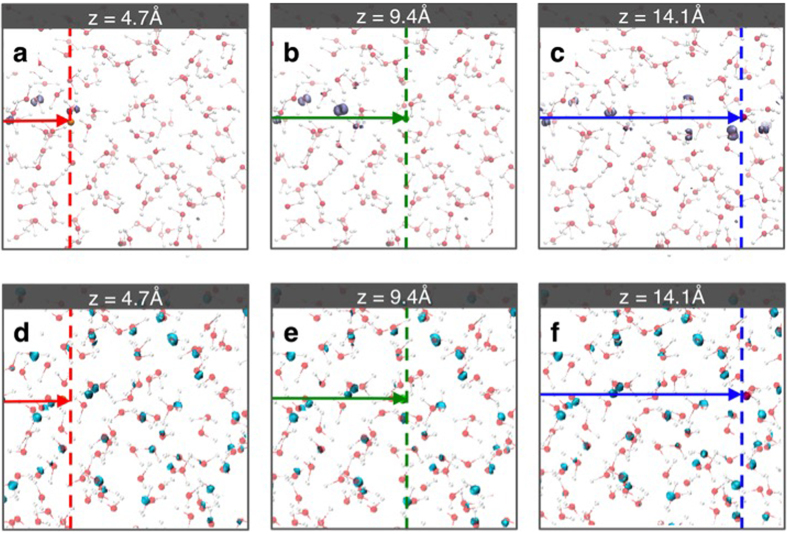
Time-dependent changes in electron density due to the valence and conduction band state occupations in response to the energetic proton’s movement. (**a**,**b**,**c**) Show the isosurface of holes generated in the valence band states at three different position of the energetic proton (4.7 Å, 9.4 Å and 14.1 Å). (**d**,**e**,**f**) Show the isosurface of the excited electrons in the conduction band states at the same three positions of the proton.

**Figure 4:**
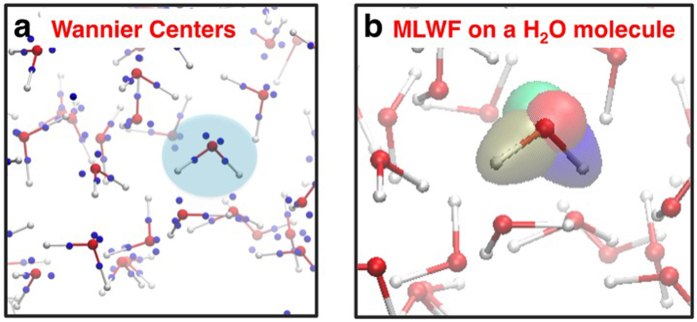
Maximally localized Wannier functions in liquid water. (**a)** Geometric centres of the maximally localized Wannier functions (blue) plotted alongside the atomic position of liquid water. (**b)** Four Wannier functions on a particular water molecule in liquid water (indicated by blue shade in (**a**)).

**Figure 5:**
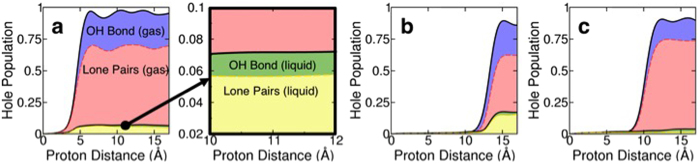
Hole populations generated on individual molecules. (**a,b**) and (**c**) show response of three different water molecules along the path of the energetic proton. The gas phase water response is given by the sum of red and blue areas where the red area represents the contribution from the lone pair electrons and the blue area represents the contribution of the OH-bonds. Liquid phase response is represented by the sum of yellow and green areas where the yellow area represents the contribution of the lone pairs and the green area represents the contribution of the OH bonds both in liquid water.

The initial stages of the water radiolysis mechanism are generally discussed in the context of electronic excitations and ionization occurring at the individual water molecule level in the literature (e.g. see Fig. 1) 18. The single-particle excitations, however, do not reveal the molecular-level details of the excitation process observed in our simulation (i.e. ionization along the proton path) because many of the eigenstates are quite delocalized over a number of water molecules even for those in the valence band. Therefore, it is insightful to characterize the excitation dynamics in terms of a spatially localized description of the equilibrium electronic structure rather than in terms of their eigenstates, for developing a conceptual understanding at the molecular level. We exploit the gauge invariance of the occupied, single-particle eigenstates and employ the maximally-localized Wannier function^27^,^28^ (MLWF) transformation. This unitary transform is performed such that the resulting single-particle wavefunctions are maximally localized in space, and four MLWFs reside on each water molecule. Of these MLWFs, two correspond to OH bonds, and other two can be identified as lone pair electrons on the oxygen atom as shown in Fig. 4. These MLWFs in the liquid water are highly localized with the average spread of ~0.49 Å^2^. For a given water molecule in the liquid water, projection of the time-dependent wavefunctions onto the corresponding four MLWFs allows us to calculate hole population changes on individual water molecules. Figure 5 shows how the hole population increases on three different water molecules in liquid water as they interact with the highly localized field of the energetic proton as it comes close to each water molecule. The MLWF occupation changes also allow us to study relative contributions of lone pairs and OH bond electrons in the ionizing excitation event on individual water molecules. Figure 5 also shows the contribution of lone pair electrons to the excitation for each of the three molecules along the proton path. Consistent amongst all the water molecules was that lone pair electrons are involved in the electronic excitation to a much greater extent than the electrons in OH bonds. The average contribution from the lone pairs is 70.1 ± 23.2%, depending on the molecules’ position and orientation along the proton path.

**Figure 6:**
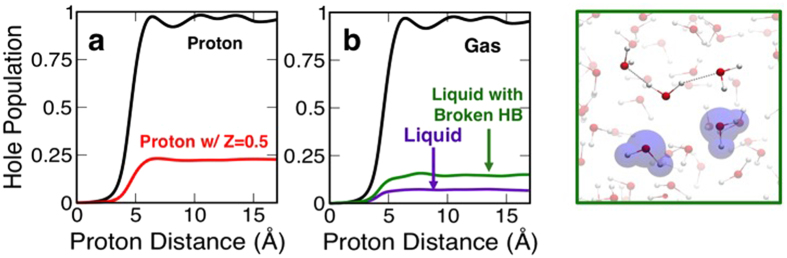
Role of effective charge state of the energetic proton and hydrogen bonding in excitation dynamics. (**a**) Comparison between proton (black) and an “artificial” proton with Z = 0.5 (red) as the energetic proton for gas phase water molecule. (**b**) Comparison for a water molecule in liquid water (purple) and liquid with two dissociated hydrogen bonds (green), and gas phase water (black). The inset shows the two water molecules (shaded purple) that are rotated such that two hydrogen bonds are dissociated, exposing lone-pair electrons of the water molecule for which the response is calculated.

We also considered this electronic stopping process for water molecules in the gas phase in order to uncover any differences with the liquid phase case. This is important because an isolated water molecule is often used to obtain parameters for analytical models in calculating the electronic stopping power^11^,^29^. In Fig. 5, the hole population changes are shown for three different water molecules in the gas phase. Their geometries and orientations are taken from the liquid water simulation for direct comparison. The excitation is significantly suppressed when the water molecule is a part of liquid water, although lone pair electrons are again involved in the excitation to a much greater extent than the OH bonds (Fig. 5). The trend of the suppressed electronic response is observed for all water molecules along the energetic proton path but to a varying degree, ranging by 80–95% in terms of the hole population. In the gas phase case, the dielectric response does not have contributions from the local field effect due to surrounding water molecules. Although analytical models of electronic stopping power suggest an important role of the dielectric function (see Method section)^11^, given the similar behaviour of the dielectric response function in the optical limit for liquid and gas-phase water^30^, it is unlikely to explain the large observed difference. At the same time, atomistic features could be at play. Linear response models such as the Lindhard formula^31^,^32^ and Bethe theory^33^,^34^ suggest that the energy transfer rate also depends quadratically on the charge state of the energetic proton in addition to the electronic response of the target matter. In condensed phase systems, the proton’s charge does not remain constant, and the concept of effective charge on the energetic ion has been discussed extensively in the literature^35^,^36^,^37^. The effective charge depends on both the proton velocity and also the medium through which the ion is traveling. Indeed, our recent first-principles quantum dynamics simulations show a significant dependence of the proton’s mean charge state on the velocity in liquid water^24^. Quantum-mechanically, determination of the charge state of the proton that is part of a larger quantum system (i.e. liquid water) depends on how the electrons’ probability density is partitioned. The charge state of the proton in liquid water is calculated roughly to be +0.5 ± 0.15 q for the kinetic energy of 20 keV, depending somewhat on the specific partitioning procedure employed and the energetic proton’s location in the liquid water (see Method section). In order to understand the extent to which the proton’s reduced charge in liquid water is responsible for the suppressed electronic response in comparison to the gas phase case, we simulated the response of a single water molecule in the gas phase with an “artificial” proton with the nuclear charge of +0.5q with no electrons. As can be seen in Fig. 6a, the proton’s reduced charge has a significant impact on the response. Indeed, the response follows roughly the quadratic dependence on the ion charge as one might expect from linear response theory.

Given an earlier work on the importance of hydrogen bonds for exciton delocalization in liquid water^38^, the role of hydrogen bonds was also considered for explaining the supressed electronic response, especially since we found that a majority of water molecule’s response derives from lone-pair electrons at the oxygen atom. In a separate liquid water simulation, two water molecules are rotated such that the two hydrogen bonds involving the lone pairs of a particular water molecule are both broken as shown in Fig. 6b. Figure 6b shows that the response of the water molecule with the broken hydrogen bonds in liquid water is much closer to the response in fully hydrogen-bonded liquid water than to the response of the gas phase water. Although the local environment of the hydrogen bonding appears to somewhat affect the electronic excitation in liquid water (e.g. the suppression is less prominent), it is not the predominant reason for the observed suppression of the response in the liquid phase. This finding is consistent with earlier work, which found that hydrogen bonds do not play a significant role in studying electronic stopping with a single water molecule in the gas phase^29^.

## Conclusion

Using large-scale first-principles quantum dynamics simulations, we studied electronic excitation dynamics in liquid water under proton irradiation. Specifically, we investigated the electronic stopping process in liquid water with the energetic proton with the kinetic energy of the maximum electronic response. Our simulations show the ionizations of individual water molecules along the proton track in the liquid water. We investigated the electronic response also at a microscopic level by employing maximally-localized Wannier functions that are localized on individual water molecules to obtain molecular-level insights. Although the molecule’s electronic excitation response largely derives from the lone pair electrons at oxygen atoms, the formation of hydrogen bonds in liquid water is not responsible for the significant difference observed between water molecules in liquid phase and in the gas phase. Instead, we have shown that the reduced charge of the energetic proton in the liquid water is responsible for the observed suppression of the electronic response in the liquid phase. This finding underscores the importance of the velocity-dependent charge state of the proton in liquid water as we reported in our recent work^24^. The present results also shed important insights into water radiolysis under proton irradiation and proton cancer therapy as a whole. Even for photo-induced water-mediated DNA damages, the exact mechanisms that lead to DNA damage are still under debate. For instance, recent works by Ngyuen *et al*. shows that pre-hydrated excited electrons cause more significant damages via reductive reaction pathways than previously believed^19^,^21^. This is in addition to the more conventional view that excited water molecules lead to radical species and thus are likely to participate in oxidative DNA damage pathways^18^. The same debate on the extent to which pre-hydrated electrons may be responsible for the water-mediated DNA damage mechanisms would apply to proton irradiation as well. Under proton irradiation, our work shows that OH• formation would not be a result of dissociation of excited water molecules but rather from the molecular ionizations (See Fig. 1). Furthermore, the electronic excitation does not induce the loss of electron density in the OH bonds but rather of oxygen atoms’ lone pair electrons. Given that the carrier relaxation of excited holes is very fast in extended systems like liquid water, dissociation of H_2_O^+^ is likely to take place after the hole has relaxed to the valence band maximum. Under proton irradiation, OH• formation is therefore likely to be a secondary process of the electronic excitation under proton irradiation via H_2_O^+^ + H_2_O → H_3_O^+^ + OH•, which is one of the two OH• formation pathways often discussed in the context of water radiolysis under photon irradiation (Fig. 1).

## Methods

### Real-Time Time-Dependent Density Functional Theory

We perform non-equilibrium simulations in which the electrons respond dynamically to an energetic proton moving at a constant velocity, using our recently-developed, highly-scalable real-time time-dependent density functional theory (RT-TDDFT) approach. Implementation details of our RT-TDDFT^39^ are discussed in refs 22 and 23. A detailed procedure of how the non-equilibrium simulations are performed are reported in ref. 25, and the reliability of this approach has been shown for liquid water in our recent work^24^. A small time step of 0.2 attoseconds was used to ensure strict convergence of our simulations. PBE exchange-correlation functional^40^ was used with adiabatic approximation in RT-TDDFT simulations^41^. A plane-wave cuttoff of 50 Ry at gamma point only in the Brillouin zone integration was found sufficient due to the large simulation cell containing 162 water molecules. Periodic boundary conditions are used, and the positions of water molecules are taken from a snapshot of an equilibrated trajectory from a 20-picoseconds first-principles molecular dynamics (FPMD) simulation^42^ at the room temperature, following ref. 43. The time-step of 10 a.u. was used for integration in the FPMD simulation. Hamann-Schluter-Chiang-Vanderbilt^44^ norm-conserving pseudopotentials were used for both hydrogen and oxygen atoms. Two different proton trajectory paths were analysed, and both of which were chosen such that the projectile/energetic proton does not penetrate into the cutoff radius of the pseudopotentials. This excluded volume from the pseudopotentials amounts to less than 5% of the total simulation cell volume.

### Maximally-Localized Wannier Function Analysis

Analysis based on maximally localized Wannier functions^27^,^28^ (MLWF) was carried out by using a modified Wannier90 code^45^ with electronic structure data from Q-box code^46^ with the above RT-TDDFT implementation. We first find the unitary transformation matrix that generates the MLWF from the valence band states at t = 0 (equilibrium ground state electron density of liquid water). Then, we use the unitary matrix and the projection of the time-dependent Kohn-Sham states onto the eigenstates in the valence band (t = 0) to determine the extent to which each Wannier function participates in the electronic excitation for individual water molecules.

### Charge State of Proton in Liquid Water

To calculate the mean charge state of the projectile/energetic proton in liquid water, two different approaches were used to quantify the amount of electron density that surrounds the energetic proton as it passes through liquid water. We first used a Voronoi partitioning scheme^47^ to identify the electron density volume centred around the proton. We integrate the electron charge density within the volume at ten evenly spaced time steps. The volume of the Voronoi cell associated with the proton changes as it moves through simulation cell, yielding a finite distribution for the effective charge. This approach has been used in our recent work^24^. Another approach we employ is motivated by the numerical work by Arista and Lifschitz as discussed in refs 48,49 in which the projectile ion charge after exiting a solid was found to be close to the mean ion charge within the solid. We quantify the amount of electron density around the proton in the vacuum right after it travels through a 8 Å slab of liquid water using Bader decomposition^50^. The analysis is performed when the proton is sufficiently far away from the water slab such that the Bader volume does not overlap with the water slab. The Voronoi approach and Bader decomposition approach give the mean proton charge of 0.51q ± 0.12q and 0.6q for the kinetic energy of 20 keV (v = 0.9 a.u.), respectively. In order to quantify the extent to which the reduced charge state of ~0.5q on the energetic proton is responsible for the suppression of electronic excitation in the liquid phase, we modelled the electronic stopping process with a water molecule in the gas phase using a fictitious energetic proton with Z = 0.5q (instead of Z = 1.0q) by modifying the proton pseudo-potential but keeping its form the same.

### Analytical Models of Electronic Stopping

A widely used analytical model of electronic stopping is based on linear response theory. Lindhard^31^,^32^ derived the stopping power (energy transfer rate per unit particle distance) as


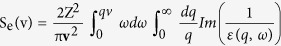


where *v* is the velocity of the projectile ion, Z is the ion charge, and ε is the macroscopic dielectric function of frequency ω and momentum transfer q. Another widely-used model, Bethe theory, also shows the same quadratic dependence on the ion charge but only the optical limit (*q* = 0) of the dielectric function enters into the equation^33^,^51^,^52^.

## Additional Information

**How to cite this article**: Reeves, K. G. and Kanai, Y. Electronic Excitation Dynamics in Liquid Water under Proton Irradiation. *Sci. Rep.* **7**, 40379; doi: 10.1038/srep40379 (2017).

**Publisher's note:** Springer Nature remains neutral with regard to jurisdictional claims in published maps and institutional affiliations.
